# Melioidosis in Papua New Guinea and Oceania

**DOI:** 10.3390/tropicalmed3010034

**Published:** 2018-03-15

**Authors:** Jeffrey M. Warner, Bart J. Currie

**Affiliations:** 1 Australian Institute of Tropical Health and Medicine, James Cook University, Townsville 4811, Australia; 2 College of Public Health, Medical and Veterinary Sciences, James Cook University, Townsville 4811, Australia; 3 Global and Tropical Health Division, Menzies School of Health Research, Charles Darwin University, Darwin, NT 0815, Australia; Bart.currie@menzies.edu.au; 4 Department of Infectious Diseases, Royal Darwin Hospital, Darwin, NT 0815, Australia

**Keywords:** Papua New Guinea, Oceania, melioidosis

## Abstract

Melioidosis has only been sporadically reported throughout Melanesia and the Pacific region since the first report from Guam in 1946; therefore, its contribution to the disease burden in this region is largely unknown. However, the outcome of a small number of active surveillance programs, serological surveys, and presumptive imported cases identified elsewhere provide an insight into its epidemiology and potential significance throughout the region. Both clinical cases and environmental reservoirs have been described from the rural district of Balimo in the Western Province of Papua New Guinea and from the Northern Province of New Caledonia. In both these locations the incidence of disease is similar to that described in tropical Australia and *Burkholderia pseudomallei* isolates are also phylogenetically linked to Australian isolates. Serological evidence and presumptive imported cases identified elsewhere suggest that melioidosis exists in other countries throughout the Pacific. However, the lack of laboratory facilities and clinical awareness, and the burden of other infections of public health importance such as tuberculosis, contribute to the under-recognition of melioidosis in this region.

## 1. History

**Figure 1:**
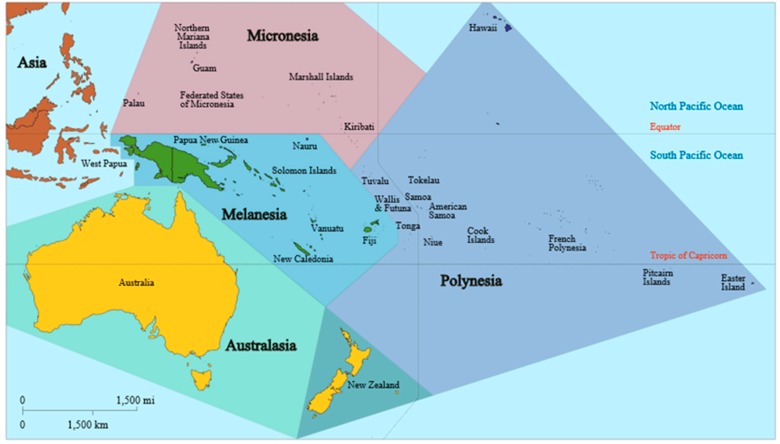
Map of Oceania based on the United Nations geoscheme M49 coding classification, devised by the United Nations Statistics Division.

The Western Pacific is home to the island communities of Oceania, including 15 countries within Melanesia, Micronesia, Polynesia, and Australasia (Figure 1). Each of these countries has diverse geography, demography, and socio-economic status. Melioidosis within Australasia will not be discussed in this review. 

The first reported cases of melioidosis in Oceania outside Australasia were from two military personal stationed on Guam during World War II [1]. In Papua New Guinea (PNG), melioidosis seems sporadic. The first reports were from animals kept in a zoo in the urban capital of Port Moresby in 1963 [2,3,4]. The first human case from PNG was recorded in 1965 from a patient referred to Port Moresby General Hospital from Gemo Island, the then-leprosarium [5]. A case of melioidosis reported from Sydney was reported in a man with a history of World War II service in PNG [6]. Similarly, Kingston, a year later, reported from the same repatriation hospital a case of presumptive reactivation illness from an individual 24 years after World War II military service in Milne Bay, PNG [7]. In New Caledonia, *Burkholderia pseudomallei* was first isolated from soil as part of an animal health program in 1984 [8] and the first human case was reported in 1999 [9].

## 2. Review of Confirmed Human Melioidosis Cases

### 2.1. Melioidosis Is Rare in PNG’s Urban Capital

Since PNG independence in 1975, seven cases of culture-confirmed melioidosis have been reported from the PNG capital Port Moresby as a result of active case detection [10,11,12,13]. De Buse and colleagues reported a five-year-old boy presenting with pyrexia of unknown origin (PUO), who failed to respond to malaria treatment. Two from three blood cultures taken revealed *Staphylococcus aureus*. During exhaustive clinical work-up, *B. pseudomallei* along with *S. aureus* was recovered from a pus-filled nodule excised from the abdominal wall. Subcutaneous abscesses appeared which, upon culture, grew *B. pseudomallei*. Lee and Naraqi undertook a retrospective study in a review of Gram-negative pneumonia presenting in adults at Port Moresby General Hospital between February 1977 and May 1979. Of the 3500 adult in-patients during this time, 550 had been admitted with acute pneumonia. Of the 80 patients with Gram-negative bacteraemia, four were found to have primary pneumonia caused by *B. pseudomallei*. One of the cases was melioidosis pneumonia with left side consolidation and cavitation resembling pulmonary tuberculosis. The patient was a 36-year-old female who presented acutely ill with fever, cough, and pleuritic chest pain. She had a past history of tuberculosis but no other traditional co-morbidity factors were described.

Melioidosis was included in a prospective study of adult atypical pneumonia at Port Moresby General Hospital over a ten-month period, reported in 1991 [14]. Of the 175 adults admitted to the study, none revealed evidence of melioidosis using culture or indirect haemagglutination (IHA) serology. Currie described a report of melioidosis in PNG presenting in a 28-year-old highland man residing in Port Moresby with fulminating pneumonia [12]. The patient had a history of excessive alcohol consumption, smoking, and glucose intolerance; he died a day after admission. 

Between 2000 and 2002, laboratory surveillance for *B. pseudomallei* at the Port Moresby General Hospital Pathology Department and at the Central Public Health Laboratory, Port Moresby, was undertaken. From 2285 blood cultures tested from patients at Port Moresby General Hospital, two (0.09%; 95% CI 0.01–0.32%) were positive for *B. pseudomallei*. In an attempt to determine the prevalence of melioidosis in the tuberculosis (TB) patient cohort in Port Moresby, 1309 sputum samples from 529 patients were selectively cultured for *B. pseudomallei*; only 1/1309 was positive for *B. pseudomallei* [13]. These studies confirm that melioidosis does exist in the urban capital but it is rare, despite the large and increasing population and increasing numbers with risk factors such as diabetes and hazardous alcohol use [15].

### 2.2. Melioidosis Hotspot: The Balimo Region of the Western Province, PNG

In contrast to the rare reports of melioidosis in Port Moresby, a focus of endemicity has been reported and studied in the remote Balimo region of the Western Province. Following an unpublished clinical report of melioidosis in this community in 1983 [16], two periods of active case detection were conducted as part of a broader development of microbiology facilities in the 1990s. Over an 18-month period this resulted in the diagnosis of eight culture-confirmed cases [17]. A feature of the disease in this region is childhood predilection with chronic presentation, which can mimic TB. Sero-epidemiology revealed a seroprevalence in some village communities as high as those in regions in northern Australia. In the environment, particularly sites that children frequent, *B. pseudomallei* was isolated (in 2.6% of 274 soil samples overall) [18]. Of 13 clinical and 26 environmental isolates analysed, only three multi-locus sequence type (MLST)-derived genotypes have been described—the most common being ST267, with the others (ST667 and ST668) differing from ST267 by only one SNP in the *ace* gene [19]. Further phylogenetic analysis shows the origins of these isolates to be in the Australian clade, yet some characteristics of the Asian isolates are also evident. It has therefore been proposed that the ancient land mass that joined Australia and New Guinea 20,000 years ago during the last ice age provided a land bridge, which may have facilitated movement of ancestral stains of *B. pseudomallei* from Australia to Asia via PNG [19].

Since the late 1990s, further cases of suspected melioidosis from Balimo District Hospital have been noted and some have been confirmed with culture of *B. pseudomallei*. However, the majority go unreported due to a downgrading of laboratory and clinical facilities over recent decades in Balimo as in many other rural locations in PNG. This includes no resident medical staff being present in Balimo and only sporadically-available bacteriological culture facilities. In this community the emergence of multidrug-resistant tuberculosis has also complicated the clinical diagnosis of melioidosis. Only when the diagnostic capacity for infectious diseases is improved will the true burden of melioidosis be realised and managed in this community.

### 2.3. Elsewhere in Oceania

Since the first description in Guam, melioidosis elsewhere in Oceania has been equally sporadic and reports are opportunistic. Several cases in Australia have been attributed to infection acquired in Fiji [20,21]. There is seroprevalence evidence from East Timor [22], but to date, no confirmed culture of *B. pseudomallei*. Only in New Caledonia has melioidosis been more thoroughly studied and reported.

Similar to in PNG, the first melioidosis studies in New Caledonia were conducted and reported by animal health workers [8]. The first human case was reported in 1999 [9]; since then, 19 melioidosis cases have been reported and studied [23,24,25]. As seems the case in PNG, melioidosis in New Caledonia is mostly restricted to one regional province, although whether there are biogeographic, demographic, or simply healthcare access factors at play can be only determined through a thorough and structured epidemiological and environmental study across the dispersed islands of that nation. As in Australia and other endemic regions, the most common presentation is community-acquired pneumonia, which may mimic TB, yet 32% presented with chronic disease including skin lesions.

As is the case in the Balimo region of PNG, there is evidence in New Caledonia of a persistent, dominant clonal strain as defined by MLST, with ST292 being the most common in the highly-endemic east coast region. All isolates analysed with MLST were shown to cluster—along with the other isolates from Melanesia—within the Australian clade rather than the Asian clade [25].

In recent years there has been a cluster of fatal melioidosis cases on the island of Yap, located in the Federated States of Micronesia (Figure 1) [26,27]. This is currently being investigated by the US Centers for Disease Control.

## 3. Current Recommendations and Availability of Measures against Melioidosis

The studies of melioidosis from isolated communities within this region highlight the difficulties in diagnosing and treating melioidosis without appropriate laboratory and clinical facilities. It is likely—as it almost certainly remains the case today in Balimo—that the disease carries very high mortality in such circumstances. In many of these communities, the gold-standard parenteral antibiotics meropenem and ceftazidime are not available. In these communities, cotrimoxazole or even chloramphenicol may be the only available antibiotics for therapy of suspected or confirmed melioidosis.

## 4. Awareness of Melioidosis, and Current and Future Challenges

Melioidosis is not a notifiable disease in any country in Oceania outside Australasia; as a result, the general awareness of the disease in this region is poor. With such limited data and such poor ability to obtain quality data, it is therefore unwise to generalise regarding the significance of melioidosis in Oceania. However, the evidence so far gathered and reported suggests that melioidosis is rare in urban centres, where microbiology facilities are able to detect the organism, but is likely to remain undiagnosed in some rural communities that lack the healthcare systems needed to identify it. With the development of an enhanced rural medical workforce plus laboratory facilities, perhaps including the introduction of non-culture-based detection methods that do not rely on extensive laboratory expertise, this may change. However, the burden of other diseases of public health importance such as multidrug-resistant TB, may yet overwhelm these developing health systems. 

The spatial clustering of clinical melioidosis linked to parameters of the environmental reservoir, the diversity of the geography, and the limited ability to diagnose infection in this region, support the need for the establishment of a systematic remote sensing study to predict, identify, and locate regions that may harbour unrevealed melioidosis. Until these studies are conducted, melioidosis will remain an under-recognised, enigmatic condition, with high mortality rates.
